# Short-Latency Afferent Inhibition Modulation during Finger Movement

**DOI:** 10.1371/journal.pone.0060496

**Published:** 2013-04-04

**Authors:** Michael J. Asmussen, Mark F. Jacobs, Kevin G. H. Lee, Christopher M. Zapallow, Aimee J. Nelson

**Affiliations:** 1 Department of Kinesiology, University of Waterloo, Waterloo, Canada; 2 Department of Kinesiology, McMaster University, Hamilton, Canada; McGill University, Canada

## Abstract

When somatosensory input via electrical stimulation of a peripheral nerve precedes a transcranial magnetic stimulation (TMS) pulse over the primary motor cortex (M1) the corticospinal output is substantially reduced, a phenomenon known as short-latency afferent inhibition (SAI). The present study investigated SAI during rest and during pre-movement, phasic and tonic components of movement. Participants were required to perform an index finger flexion reaction time task in response to an auditory cue. In a series of experiments, SAI was evoked from the mixed, median nerve at the wrist or the cutaneous, digital nerve stimulation of the index finger. To assess the spinal versus cortical origin of movement-related modulation of SAI, F-wave amplitudes were measured during rest and the three movement components. Results indicated that SAI was reduced during all movement components compared to rest, an effect that occurred for both nerves stimulated. Pre-movement SAI reduction was primarily attributed to reduced cortical inhibition, while increased spinal excitability additionally contributed to reduced SAI during tonic and phasic components of movement. SAI was differentially modulated across movement components with mixed but not cutaneous nerve stimulation. These findings reveal that SAI is reduced during movement and this reduction begins as early as the preparation to move. Further, these data suggest that the degree of SAI reduction during movement may be specific to the volume and/or composition of afferent input carried by each nerve.

## Introduction

Somatosensory input modulates M1 excitability over a short and long time course. Short term increases or decreases in M1 excitability are evoked following stimulation of the primary somatosensory cortex (SI) in monkeys [1]. Tetanic electrical stimulation of SI causes long term changes in the excitability of the primary motor cortex (M1) in cats [2]. Somatosensory afference may also influence M1 excitability [3] via direct thalamocortical projections to M1 [4] or via a relay through SI [5], [6]. With the exception of area 3b, SI has direct connections to M1 [7].

In awake humans, the corticospinal output from M1 to intrinsic hand muscles evoked by a transcranial magnetic stimulation (TMS) pulse is substantially reduced when preceded by peripheral nerve stimulation at a short latency (∼20 ms), an effect known as short-latency afferent inhibition (SAI) [8]. The pathway mediating SAI is considered to be of cortical origin, as there is a reduction in the amplitude of later indirect waves (I-wave) [8] that are thought to represent local interneuronal or corticocortical inputs to the corticospinal output neurons in M1 [9]. SAI which occurs with both mixed and cutaneous nerve stimulation [8], is also non-selective for muscles of the hand when the mixed nerve is stimulated [10] but may show somatotopic effects for cutaneous nerve stimulation [11], [12]. SAI is dependent on the intensity of the conditioning and test stimuli [10], [13] and the size of the receptive field such that when the cutaneous nerve is stimulated for three digits SAI is decreased compared to single digit stimulation [14]. SAI is mediated by cholinergic inputs and is reduced or abolished in the presence of acetylcholine (Ach) blockers [15]. GABA modulates SAI such that GABA_A_ agonist lorazepam causes an inhibition of Ach and reduces SAI [16]–[18]. Which sub-unit is involved in this reduction is still unknown though it is suggested that the alpha-1 subunit might be involved in the decrease of SAI while the alpha-5 subunit of the GABA_A_ receptor may be implicated in the increase of SAI [16], [18], [19].

The magnitude of SAI is modifiable. SAI has been shown to interact with a number of other inhibitory circuits such that short-interval intracortical inhibition (SICI), long-interval intracortical inhibition, and short-interval inter-hemispheric inhibition all reduce SAI [20]–[22]. Movement also modifies the magnitude of SAI. Tonic muscle contraction of the first dorsal interosseous (FDI) muscle substantially reduces SAI from mixed nerve stimulation [13] and phasic contraction of FDI reduces SAI from cutaneous nerve stimulation in some instances [23] but not others [24]. These data suggest that SAI may be sensitive to the phase of the movement. However this suggestion remains inconclusive given that the intensity of the test stimulation in the tonic [13] and phasic [23], [24] components were different across these studies. Recently it has been shown that SAI may be a marker for functional recovery from stroke and may provide insight into the mechanism of stroke recovery [25]. As with most studies measuring SAI in patient populations, this study was performed at rest, but it is unknown exactly how SAI is modulated during different movement components. A thorough analysis of SAI during movement in a healthy population is a precursor for studying clinical populations, where SAI may also be abnormally altered during movement.

It is evident that SAI is reduced during movement [13], [23] though it is unknown how early in time this modulation begins. In humans there are local changes in M1 inhibitory circuits, such as SICI, as early as the delay period between a ‘warning’ and ‘go’ cue [26], [27]. In monkeys, the response amplitude of the afferent input in both the premotor cortex and M1 is reduced during the same delay period before the upcoming movement, and increased afferent gating (i.e., reduction in afferent input amplitude) coincides with faster reaction times [28]. In humans, somatosensory evoked potentials (SEPs) are reduced (i.e., gated) during the phasic and tonic components of movement [29] when SAI is also reduced [13], [23], [24], suggesting that a reduction of afferent input reaching the cortex may also coincide with less SAI (i.e. less inhibition). It remains unclear whether SAI is altered during the delay period between a ‘warning’ and ‘go’ cue and if such modulation depends on the submodality of somatosensory input, as median nerve stimulation would carry a larger volume and different content relative to the digital nerve that is predominantly cutaneous [11], [30], [31].

The purpose of this study was to determine whether SAI is modulated during the pre-movement (between ‘warning’ and ‘go’ cue), phasic (onset of muscle activity), and tonic (sustained muscle activity) components of movement, and to determine if such modulation depends on the submodal input used to elicit SAI. To test this, SAI was measured during a simple reaction time task involving phasic and tonic index finger flexion. SAI was evoked from the median nerve and also via index digital nerve stimulation. To assess the cortical versus spinal origin of the phase-dependency of SAI spinal excitability was measured using F-waves. We hypothesized that SAI would be reduced during all three movement components compared to rest. Specifically, we expected a reduction in SAI prior to movement since afferent input is shown to be reduced during this pre-movement component [28]. Further, we hypothesized that compared to the pre-movement component SAI would be further reduced in phasic and tonic components because of increased gating and increases in spinal excitability during these movement phases.

## Methods

### Ethics Statement

This study was approved by the Office of Research Ethics at the University of Waterloo and conformed to the *Declaration of Helsinki*. Written informed consent was obtained from all participants in the study.

### Participants

Thirty-seven healthy subjects (*X̄*_age_ = 25.4, SD = 5.0, 25 males) participated. From this subject pool, some individuals participated in more than one study. Two participants took part in all experiments, three participants completed Experiments 1, 3 and 4, one participant completed Experiments 1 and 4, one participant took part in Experiments 1 and 3, and one participant completed both Experiments 1 and 2. For SAI and F-wave experiments we aimed to collect ten and seven participants, respectively, as used previously [8], [23]. All participants were deemed to be right handed as per a modified version of the Edinburgh Handedness Inventory [32].

### Electromyography (EMG)

Surface silver-silver chloride EMG electrodes were placed on the skin overlying the first dorsal interosseous (FDI) muscle and the metacarpophalangeal joint of the right hand in a muscle belly-tendon montage. The analog signal from the electrodes was amplified with a gain of 1000, band-pass filtered between 20 and 2500 Hz (Intronix Technologies Corporation Model 2024F, Bolton, Ontario, Canada), and sampled at a frequency of 5000 Hz using an analog-to-digital interface (Power 1401, Cambridge Electronic Design, Cambridge, UK). The EMG electrodes were used to measure the peak-to-peak amplitude of the motor evoked potential (MEP) recorded from FDI of the right hand. Analysis was completed off-line on a personal computer using Signal software (Cambridge Electronic Design, Cambridge, UK).

### Peripheral Nerve Stimulation (PNS)

Peripheral nerve stimulation was achieved with 200 μs square wave pulses delivered via a Grass SD9 Telefactor stimulator (Grass Technologies, West Warwick, USA). The right ulnar or median nerve was stimulated at the wrist with the cathode proximal to the anode and the anode positioned ∼8 cm proximal to the thenar muscles. The ulnar nerve was stimulated at 25% higher than the minimum stimulator intensity required to evoke a maximal motor response in FDI muscle and was used to evoke F-waves [33]. The median nerve was stimulated at motor threshold defined as the lowest stimulator intensity to produce a slight twitch in the thenar muscles of the right hand, an intensity used to evoke SAI in past work [8]. The digital nerve of the index finger was stimulated using ring electrodes with the cathode proximal to the anode and positioned around the proximal and intermediate phalynx. The digital nerve was stimulated at ∼2 times perceptual threshold, an intensity shown to evoke SAI at rest [8].

### Transcranial Magnetic Stimulation (TMS)

TMS was delivered using a custom built 50 mm diameter figure-of-eight branding coil connected to a Magstim 200^2^ stimulator (Magstim, Whitland, UK). The position and orientation of the coil was monitored throughout the experiment using Brainsight Neuronavigation (Rogue Research, Montreal, Canada) with optical sensors placed on the coil and the participant. The TMS coil delivered a monophasic pulse over the optimal location to elicit MEPs in the relaxed right FDI at 45° in relation to the parasagittal plane to induce a posterior-lateral to anterior-medial current in the cortex and preferentially activate corticospinal neurons trans-synaptically [34]. The resting motor threshold (RMT) was defined as the lowest stimulator intensity to produce MEPs in the FDI of at least 50 μV in 5 out of 10 consecutive trials [35].

### Task Preparation

**Figure 1:**
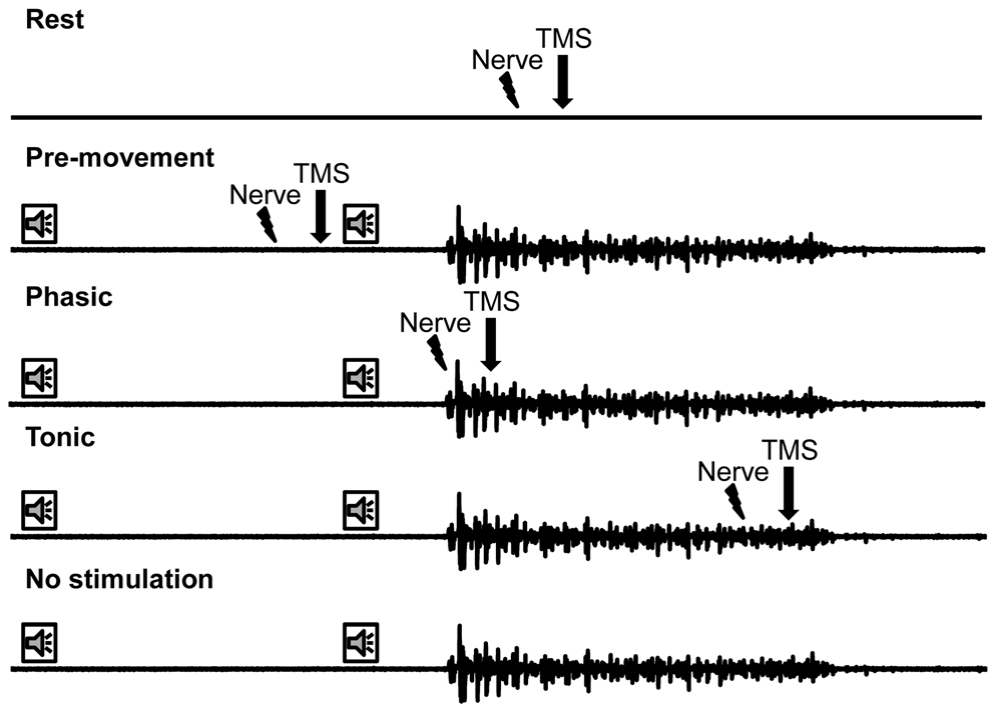
Task conditions. The timeline of the rest, pre-movement, phasic, tonic, and no stimulation conditions. The first and second speaker icons represent the ‘warning’ and ‘go’ cue, respectively. The timing of the nerve stimulation and TMS pulse are shown schematically.

An identical behavioural task was performed in all experiments. Participants performed a simple reaction time task with the response being an isometric index finger flexion to 10% of their maximum force (F_max_). To determine 10% F_max_ participants were seated with their right arm relaxed with their shoulder abducted ∼20° and elbow flexed at ∼90°. In this position, participants voluntarily flexed their index finger at the metacarpophalangeal joint maximally against a load cell (Transducer Techniques, model THA-50-Q load cell). Once the maximal force was identified, participants practiced producing 10% of their maximal index finger force (10% F_max_) using visual feedback of their force displayed on an oscilloscope. Participants were given at least 5 practice trials in which the experimenter inspected whether they could reach 10% F_max_ quickly and without substantially over or undershooting the desired force level. If the participant needed more trials to obtain this level of success, more training trials were given. For the simple reaction time task, each trial consisted of an auditory tone that served as the ‘warning’ cue followed 3 to 5 seconds later by a second auditory tone that served as the ‘go’ cue (*Figure 1*). Upon hearing the ‘go’ cue, participants flexed the index finger to 10% F_max_ with the emphasis on speed and held this contraction until instructed by the experimenter to release their force and return to the initial resting state. The voltage from the load cell was passed through a strain amplifier and the force level was displayed on an oscilloscope as a bright line. Subjects were required to position one line, representing their current force level, over a second line that marked their 10% F_max_.

### Experiment 1: Median nerve SAI

Fourteen subjects participated. SAI was investigated in four conditions: rest, pre-movement, phasic and tonic. During the rest trials, participants were required to relax their hand completely. The experimenter monitored the EMG level rejecting any trials in which there was EMG peak-to-peak amplitude >20 μV before the TMS pulse. In the pre-movement trials, a single TMS pulse was delivered one second before the go cue (*Figure 1*) and trials were rejected when there was EMG peak-to-peak amplitude >20 μV two seconds before the TMS pulse. For phasic trials, the TMS pulse was delivered after the ‘go’ cue and was computer-controlled using a sequencer file in Signal software such that the TMS pulse would automatically be triggered when the EMG from FDI reached a 100 μV threshold. In the tonic trials, the TMS pulse was delivered once the participant consistently held the 10% F_max_ as determined visually by the experimenter monitoring the force level on the oscilloscope. The TMS pulse in the tonic condition occurred approximately 2 seconds after the participant maintained the targeted force, but was moderately varied across subjects due to differences in the time needed to obtain the required level of force across participants. In addition, we included ‘no stimulation’ trials within each block that omitted median nerve and cortex stimulation and were intended to remove any anticipatory effects of brain or nerve stimulation during performance of the trials. The movement trials and no stimulation trials were presented randomly in two blocks of 40 trials that were separated by a 2 minute break. The inter-trial interval was randomized to occur between 7 and 9 seconds. The rest trials, also repeated twenty times, were completed either before or after the two blocks of 40 trials, an order that was counterbalanced across participants. The rest trials were isolated from the movement trials to ensure that testing was performed in the absence of the task to eliminate any movement preparation effects, similar to that performed elsewhere [23], [36]. Within the twenty trials for each movement component (i.e., rest, pre-movement, phasic and tonic) ten trials delivered TMS pulses only (i.e. unconditioned MEP) and the other ten delivered stimulation to the median nerve 22 ms prior to the TMS pulse to evoke SAI (i.e. conditioned MEP) [13]. Conditioned and unconditioned trials were presented randomly. The stimulator output was adjusted to elicit a MEP of ∼1 mV in each individual movement component: rest, pre-movement, phasic and tonic. To achieve this, the stimulator output required to evoke an average of ∼1 mV MEP across ten trials for each movement component was determined during the experimental set-up and this intensity was kept constant throughout the testing trials. There was no adjustment of the TMS stimulator output on a trial to trial basis because the running average of MEPs during the movement components could not be determined online. Prior to beginning the testing trials, practice trials for each movement condition were performed to allow participants to familiarize themselves with the TMS and nerve stimuli in the context of performing the task.

### Experiment 2: Digital nerve SAI

Fifteen subjects participated in Experiment 2. Experiment 2 was identical to Experiment 1 with the exception that the digital nerve of the right index finger was stimulated. The TMS pulse was delivered 25 ms following digital nerve stimulation to elicit SAI [37].

### Experiment 3: Spinal excitability during different components of movement

Seven subjects participated in Experiment 3. F-waves were evoked in FDI by stimulating the ulnar nerve at the wrist. A total of 320 ulnar nerve stimuli were delivered throughout the course of this experiment –80 stimuli for each movement component outlined in Experiment 1 (i.e., rest, pre-movement, phasic, tonic). The average of the first twenty F-waves ≥20 µV were used for the analysis as performed elsewhere [38]. We delivered 80 stimuli because the persistence of F-wave appearance is approximately 70% for the ulnar nerve [33] and since an F-wave may not appear on every trial, at least 60 electrical stimulations are needed to obtain at least 20 F-waves for averaging [33]. F-waves are a suitable measure for assessing spinal excitability in intrinsic hand muscles [33], where an H-reflex is difficult to produce. The F-waves were elicited at the identical time points as shown by the word ‘TMS’ in *Figure 1*. Similar to Experiments 1 and 2 the rest trials were presented before or after the movement trials and counterbalanced across subjects.

### Experiment 4: Mixed nerve SAI with TMS at 1.2 RMT

Twelve subjects participated. Experiment 4 was identical to Experiment 1 with the exception of the TMS intensity. The intensity of the TMS pulse was set to 1.2 RMT in all movement components (i.e., rest, pre-movement, phasic, tonic). Past research has demonstrated 1.2 RMT versus ∼1 mV normalization yielded similar results when comparing rest to tonic contraction [13]. Experiment 4 investigated whether the 1.2 RMT methodology yielded similar results as Experiment 1.

### Statistical analysis

SAI was expressed as a ratio of the peak-to-peak amplitude of the conditioned MEP (TMS pulse plus nerve stimulation) to the peak-to-peak amplitude of the unconditioned MEP (TMS pulse only). If SAI did not exist at rest (i.e., the ratio of the conditioned to unconditioned MEP was ≥1) the participant's data was not included in subsequent analyses. Spinal excitability was determined by the peak-to-peak amplitude of the F-wave.

All experiments used a repeated measures ANOVA with factor PHASE (rest, pre-movement, phasic, tonic). The hypotheses of reduced SAI during the components of movement were tested using *a priori* dependent samples *t*-tests to determine differences between individual components of the movement (i.e., rest versus pre-movement, tonic and phasic; pre-movement versus phasic and tonic). If a significant main effect of PHASE was found for spinal excitability, Tukey's post hoc analysis was performed. Sphericity was tested for all of the repeated measures ANOVA and when this assumption was violated the Greenhouse Geisser correction was implemented. For all statistical tests, the alpha level was set at *p*≤0.05.

Aside from the main analyses, a number of additional analyses were performed. First, changes in unconditioned MEP amplitude may lead to changes in SAI [13]. For this reason, to determine if there was a difference in the average peak-to-peak amplitude of the unconditioned MEPs across the different movement components, a repeated measures ANOVA with the factor PHASE, followed by a post hoc Tukey's test was performed on the unconditioned MEP amplitudes. Second, to determine if SAI existed in each phase, dependent samples *t*-tests were used for each component of the movement (i.e., pre-movement, phasic, tonic) by comparing the means of the conditioned and unconditioned MEP. Third, to determine if there were differences in the degree of SAI between digital versus mixed nerve stimulation in each condition (i.e., rest, pre-movement, phasic, tonic) independent samples *t*-tests were administered. Since three participants completed Experiments 1 and 2, two of the participants were removed from Experiment 1, while the other was removed from Experiment 2 for this analysis only. Last, changes in SAI during the pre-movement component may correlate with reaction time, as gating of afferent input prior to movement coincides with faster reaction times [28]. Therefore, we examined whether reaction time correlated with the degree of SAI in the pre-movement component. Reaction time was defined as the time elapsed between the onset of the ‘Go’ cue and the EMG 100 µV threshold for trials in which SAI was tested during the pre-movement component.

## Results

### Experiment 1: Median nerve SAI

**Figure 2:**
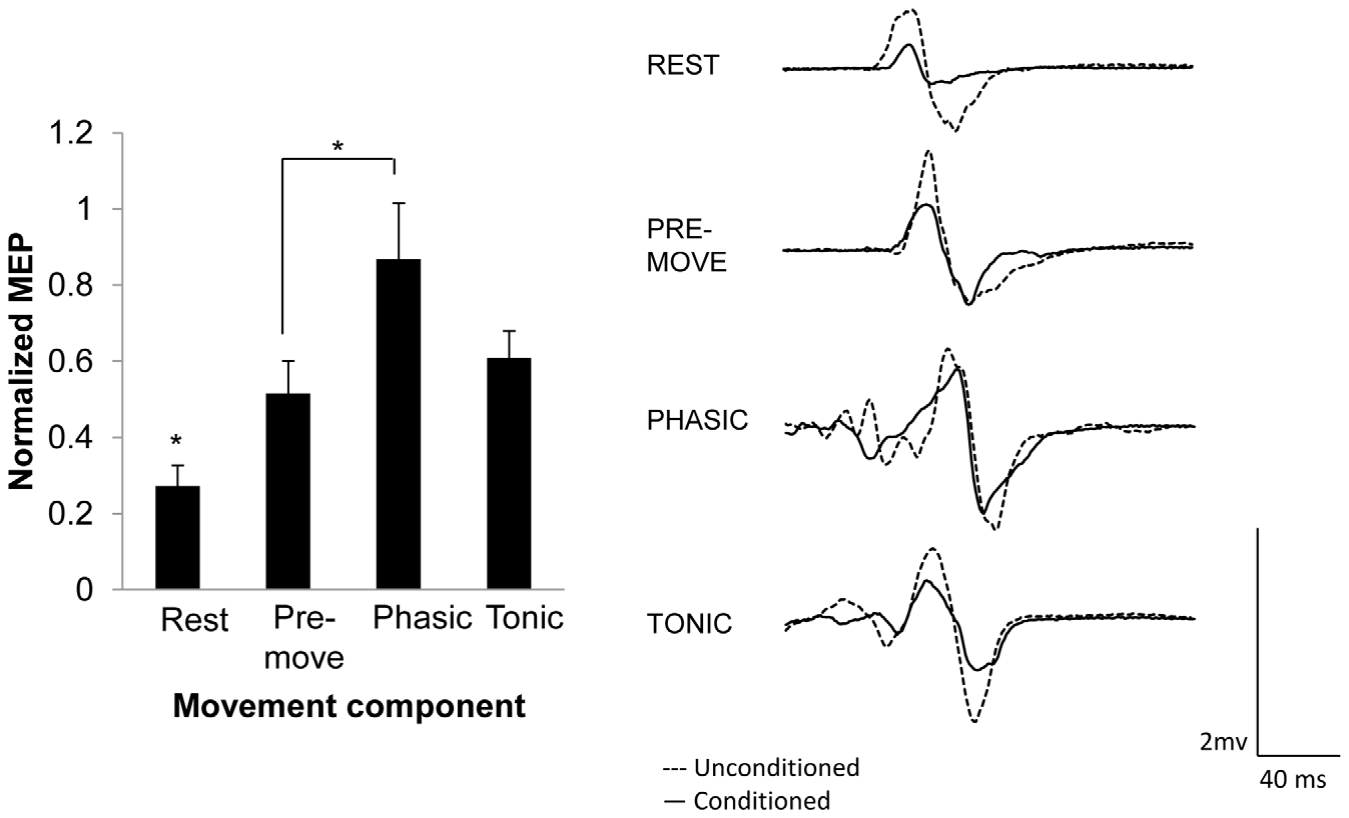
SAI induced by mixed nerve stimulation during different components of movement with TMS normalized to ∼1 mV. Left: Group-averaged data (with standard error of the mean) for rest and each component of movement. Right: individual trial EMG traces from one participant demonstrating changes in SAI across task conditions. An asterisk over a single component of movement indicates it was significantly different than all other components of the movement. An asterisk over a bar connecting two components of movement indicates those phases are significantly different. Significant differences were tested at *p*≤0.05.

Experiment 1 tested whether SAI was altered during the different components of movement using median nerve stimulation. Three participants were excluded from the study because they did not exhibit SAI at rest. The remaining eleven subjects were included in the analysis (*X̄*_age_ = 25.4, SD = 5.5, 8 males). The group average of percentage of maximal stimulator output (MSO) for the rest, pre-movement, phasic, and tonic components was 54.8%, 54.4%, 35.5%, and 42.2%, respectively. Mean 10% F*_max_* across these participants was 3.58 N±0.58. *Figure 2* displays the group-averaged data (with standard error of the mean) for each movement component. Repeated measures ANOVA revealed a significant main effect of PHASE (*F*_(3,30)_ = 7.420, *p* = 0.009). *A priori* comparisons revealed that SAI was reduced in all components of movement compared to rest (pre-movement *p* = 0.007, phasic *p* = 0.001, tonic *p* = 0.002), and comparing among the movement components, SAI was reduced in phasic versus pre-movement condition (*p* = 0.021). Paired *t*-tests revealed that SAI existed during pre-movement (*p*<0.0001), and tonic (*p* = 0.026), but not during the phasic component of movement (*p* = 0.10). Last, the peak-to-peak amplitude of the unconditioned MEPs were not different across the conditions (*F*_(3,30)_ = 0.826, *p* = 0.49) meaning that the test stimulation amplitude was successfully normalized across the conditions. To ensure that the magnitude of the unconditioned MEP amplitudes did not affect the degree of SAI, a Pearson's correlation coefficient was performed on the averaged unconditioned MEP amplitude and degree of SAI for each participant and revealed no significant correlation (*r* = −0.049, *p* = 0.754). These analyses are important since a larger unconditioned MEP could lead to reduced SAI [13] and these data indicate that changes in SAI are related to the movement component rather than the amplitude of the unconditioned MEP. Last, the average reaction time across individuals was 263±19 ms and did not significantly correlate with the degree of SAI in the pre-movement component (*r* = −0.001, *p* = 0.99). In summary, there was reduced SAI during all movement components compared to rest and in the phasic compared pre-movement component without any changes in unconditioned MEP amplitudes in each respective component.

### Experiment 2: Digital nerve SAI

**Figure 3:**
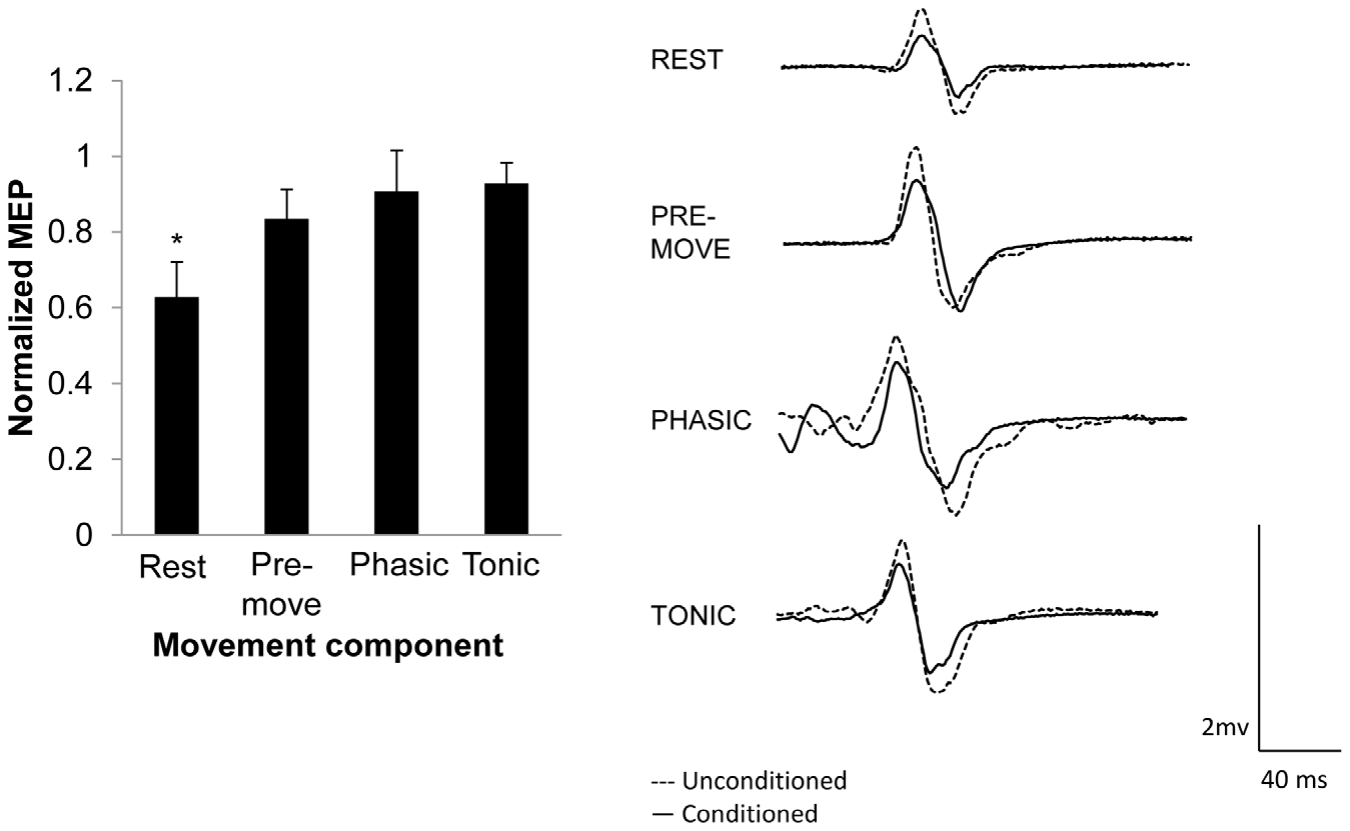
SAI induced by cutaneous nerve stimulation during different components of movement with TMS normalized to ∼1 mV. Left: Group-averaged data (with standard error of the mean) for rest and each component of movement. Right: individual trial EMG traces from one participant demonstrating changes in SAI across task conditions. An asterisk over a single component of movement indicates it was significantly different than all other components of the movement. Significant differences were tested at *p*≤0.05.

Experiment 2 tested whether SAI evoked by digital nerve stimulation was altered during the different components of movement. Four subjects did not show SAI at rest and the data from the remaining eleven subjects were used in the analysis (_age_ = 22.4, SD = 3.1, 4 females). The group average percentage of maximal stimulator output (MSO) for the rest, pre-movement, phasic, and tonic components were 57%, 56.3%, 35.1%, and 41.3%, respectively. Mean 10% F*_max_* across these participants was 3.82 N±0.39. *Figure 3* displays the group-averaged means (with standard error of the mean) for each component of the movement. Repeated measures ANOVA revealed a significant main effect of PHASE (*F*_(3,30)_ = 4.047, *p* = 0.016). *A priori* comparisons revealed that, similar to the mixed nerve, SAI was reduced in all components of movement compared to rest (pre-movement *p* = 0.04, phasic *p* = 0.02, tonic *p* = 0.004). There was, however, no difference in the magnitude of SAI between the different components of movement. In addition, the paired comparisons revealed that SAI existed during pre-movement (*p* = 0.03), but not the phasic (*p* = 0.14) or tonic (*p* = 0.18) components of movement. Last, the repeated measures ANOVA for unconditioned MEP peak-to-peak amplitude did not reveal a significant main effect for PHASE (*F*_(3,30)_ = 3.672, *p* = 0.06). To ensure that the magnitude of the unconditioned MEP amplitudes did not affect the degree of SAI, a Pearson's correlation coefficient was performed on the averaged unconditioned MEP amplitude and degree of SAI for each participant and revealed no significant correlation (*r* = −0.11, *p* = 0.478) indicating that the magnitude of the unconditioned MEPs in this range did not relate to the degree of SAI and the differences in SAI depend on the component of the movement. Last, the average reaction time across all participants was 234±19 ms and did not correlate with the degree of SAI in the pre-movement component (*r* = 0.27, *p* = 0.43). In summary SAI was reduced during all components of movement compared to rest without changes in unconditioned MEP amplitude.

### Comparison of SAI in Experiments 1 and 2

To determine if there were differences in the degree of SAI between the mixed versus cutaneous nerve stimulated in Experiment 1 and 2, respectively, independent samples *t*-tests were performed. Differences in SAI between the nerves existed such that there was less SAI, meaning less inhibition, when the cutaneous nerve was stimulated versus the mixed nerve in the rest (*X̄*_mixed_ = 0.32 vs. *X̄*_cutaneous_ = 0.60, *p* = 0.01), pre-movement (*X̄*_mixed_ = 0.55 vs. *X̄*_cutaneous_ = 0.80, *p* = 0.04), and tonic (*X̄*_mixed_ = 0.58 vs. *X̄*_cutaneous_ = 0.95, *p* = 0.001) conditions, but there were no differences between the nerves for the phasic condition (*X̄*_mixed_ = 0.90 vs. *X̄*_cutaneous_ = 0.96, *p* = 0.743).

### Experiment 3: Spinal excitability during different components of movement

**Figure 4:**
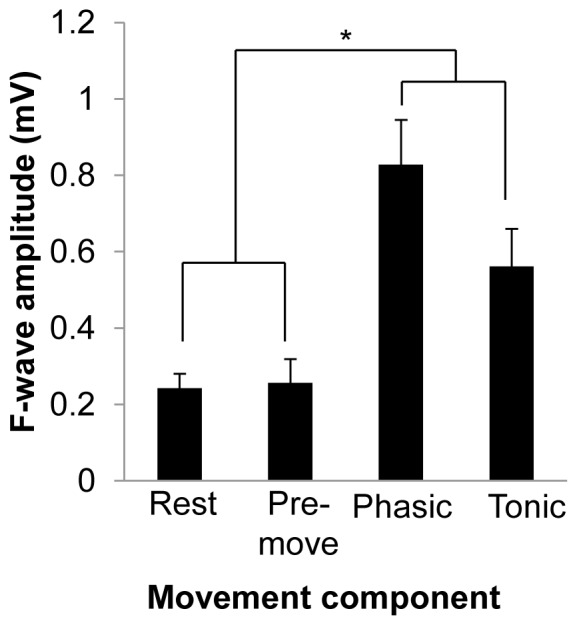
F-wave amplitude during different movement components in the index finger flexion task. Group-averaged data (with standard error of the mean) for each component of movement. The asterisk over the bar connecting the movement phases to rest and pre-movement conditions indicates that F-wave amplitude in both movement phases were significantly different than rest and pre-movement. Significant differences were tested at *p*≤0.05.

Seven subjects completed Experiment 3 (*X̄*_age_ = 25.86, SD = 2.6, 6 males) and all were included in the analysis. Experiment 3 tested the spinal excitability during the different components of movement in the FDI of the right hand using F-waves. *Figure 4* displays the group means (with standard error of the mean) for each movement component. The repeated measures ANOVA revealed a significant main effect of PHASE (*F*_(3,18)_ = 29.895, *p*<0.0001). Tukey's post hoc analysis revealed that there were significant differences between F-waves during rest versus phasic (*p*<0.0001), rest versus tonic (*p* = 0.03), pre-movement versus phasic (*p*<0.0001), pre-movement versus tonic (*p* = 0.04), but phasic versus tonic only approached significance (*p* = 0.09). These data indicate that, compared to rest, spinal excitability was increased during movement but not during the preparation to move.

### Experiment 4: Mixed nerve SAI with TMS at 1.2 RMT

**Figure 5:**
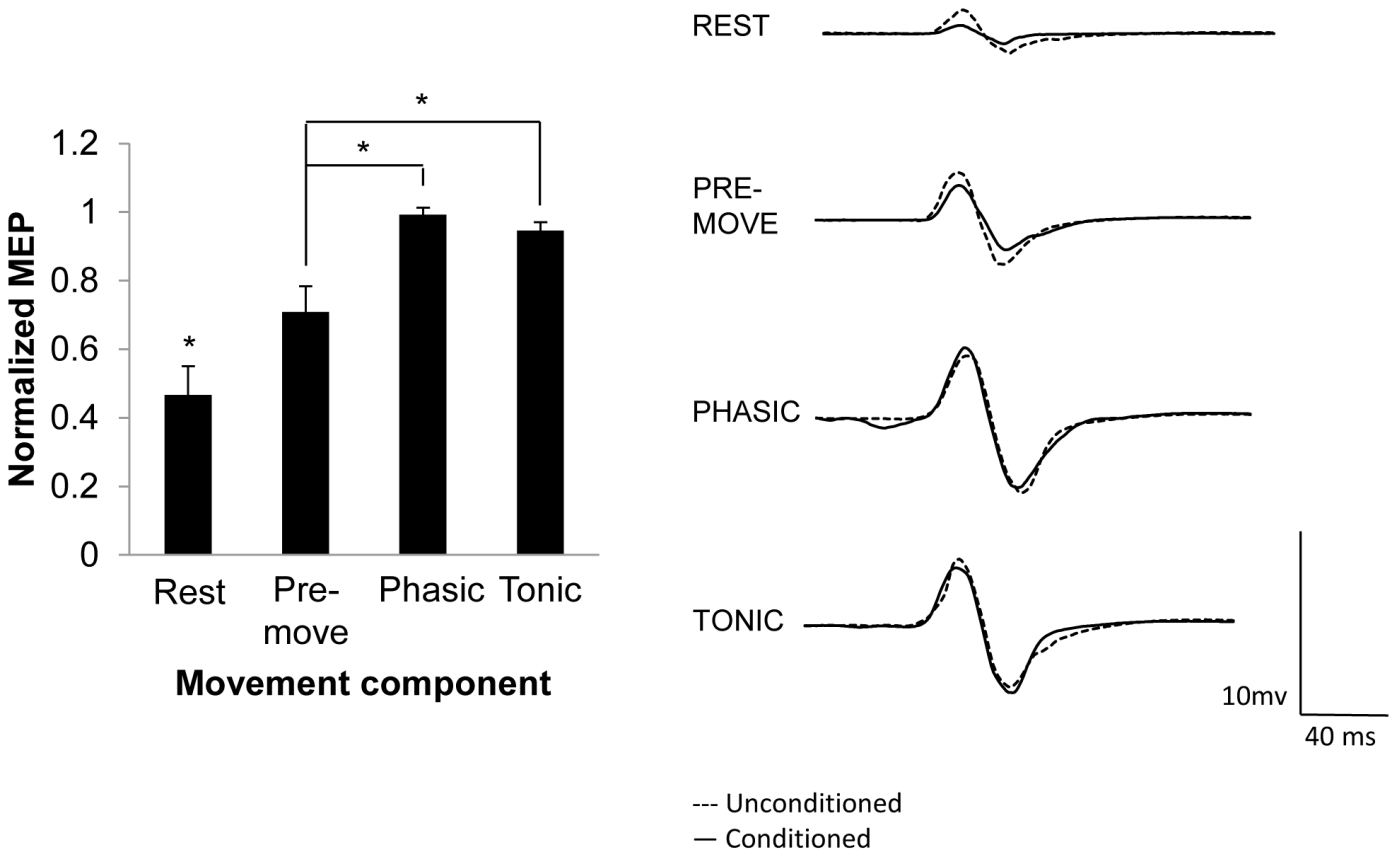
SAI induced by mixed nerve stimulation during different components of movement with TMS intensity at 1.2 RMT. Left: Group-averaged data (with standard error of the mean) for rest and each component of movement. Right: individual trial EMG traces from one participant demonstrating changes in SAI across task conditions. An asterisk over a single component of movement indicates it was significantly different than all other components of the movement. An asterisk over a bar connecting two components of movement indicates those components are significantly different. Significant differences were tested at *p*≤0.05.

This experiment tested whether SAI was altered during the different components of the movement using median nerve stimulation and a TMS intensity of 1.2 RMT. Using a 1.2 RMT normalization is technically easier to obtain across the movement components, but the problem with this approach is that the corticospinal excitability might be altered across the movement components and could potentially confound the SAI results. Three people did not show SAI at rest and were excluded from the analysis. Nine subjects were therefore included in these results (*X̄*_age_ = 28.7, SD = 5.7, 4 females). The average percentage of maximal stimulator output for the group at 1.2 RMT was 45%. Mean 10% F*_max_* was 4.18 N±0.46. *Figure 5* displays the group-averaged mean (with standard error of the mean) for each movement component. Repeated measures ANOVA revealed a significant main effect of PHASE (*F*_(3,24)_ = 21.20, *p*<0.0001). *A priori* paired comparisons revealed that, compared to rest, SAI was reduced in all components of movement (pre-movement *p* = 0.008, phasic *p*<0.0001, tonic *p* = 0.001). Further, SAI was significantly reduced during the phasic and tonic components compared to the pre-movement phase (phasic *p* = 0.006, tonic *p* = 0.017). In addition, to test for the presence of SAI during each movement component, paired comparisons revealed its existence during pre-movement (*p* = 0.004), but not during phasic (*p* = 0.99) and tonic (*p* = 0.15) components. Last, to test for differences in the amplitude of the unconditioned MEPs, the repeated measures ANOVA revealed a significant effect of PHASE (*F*_(3,24)_ = 47.52, *p*<0.0001). Tukey's post hoc analysis revealed significant differences between rest versus phasic (*p*<0.0001), rest versus tonic (*p*<0.0001), pre-movement versus phasic (*p* = 0.001), and pre-movement versus tonic (*p* = 0.003). To test whether the magnitude of the unconditioned MEP amplitudes affected the degree of SAI, a Pearson's correlation coefficient was performed on the averaged unconditioned MEP amplitude and degree of SAI for each participant. This analysis revealed a significant correlation (*r* = 0.64, *p*<0.0001) indicating that as the size of the unconditioned amplitude increased, SAI was concurrently reduced. In summary, SAI was reduced during all movement components compared to rest, similar to the results in Experiment 1 using a ∼1 mV normalization procedure. However, the reduction in SAI across the movement components may have been confounded by the increase in unconditioned MEP size during the phasic and tonic components of the movement.

## Discussion

The present study investigated the modulation of SAI in the context of movement and identified somatic inputs that drive these alterations. SAI was measured during rest and during the pre-movement, phasic and tonic components of an index finger flexion reaction time task. We observed that SAI was decreased during all movement components compared to rest. The magnitude of SAI reduction was, however, dependent on the movement component and the nerve stimulated. The data suggest that increases in spinal excitability contribute to reduced SAI during movement while reductions in SAI prior to movement appear to be primarily cortically mediated.

SAI was reduced in all components of movement regardless of the nerve being stimulated. Reduction in SAI has been shown during the phasic and tonic component for the digital nerve [11], [23], [24] and in the tonic component for the mixed nerve [13]. In our study SAI was reduced in the phasic, tonic and also the pre-movement component for both types of submodal inputs. Specifically, SAI was reduced in the phasic component of movement by 27% similar to the ∼25–30% reduction shown elsewhere [23]. Further, SAI was reduced by 30% during tonic contraction, similar to previous reports using median nerve stimulation [13], but less than the 50% reduction in SAI observed for digital nerve stimulation in past research [11]. The latter difference may relate to specific movement such that the 1^st^ and 5^th^ digit performed the tonic contraction [11].

There are several mechanisms that could mediate the reduction in SAI during the phasic and tonic components of movement. At rest, SAI is reduced with administration of GABA_A_ agonist lorazepam, suggesting that GABAergic inhibitory interneurons are mediating this reduction [16]–[18]. During movement, reduced SAI may also be mediated by somatosensory afferent gating within SI or sub-cortical loci that would result in less inhibition in M1. For example, during muscle contraction, SEPs are gated in the tonic component of movement compared to rest and further gated during EMG onset [29]. Our SAI data showed the same trend. Compared to rest, SAI was reduced in the tonic component and the reduction was even greater during the phasic component. Therefore, it appears that the magnitude of SAI may be related to the amplitude of SEPs such that an increase in SAI (i.e., more inhibition) may evoke concomitant increases in SEP amplitude (i.e. less gating) indicating an increase in activity within SI.

Changes in spinal excitability may also account for reduced SAI during movement. Spinal excitability is increased during phasic and tonic components of movement [23], [24], [39], [40] and we observed the same result. At rest, summation of three I-waves are needed to produce a MEP from a TMS pulse, while only the I1 wave is necessary to create a MEP during low voluntary contraction due to increased spinal excitability [34]. Since short-latency somatosensory input does not affect the I1 wave [8], the increased spinal excitability during the phasic and tonic components would allow the unaffected I1 wave to contribute to the MEP and yield reduced SAI in relation to rest. This evidence does not rule out the fact that the cortex may also contribute to reduced SAI during movement, as the number of I-waves produced during voluntary contraction increases [41]. However, the amplitude of I-waves are unaltered at 20% MVC [41], a similar force level used in the present study, therefore we suggest that increases in spinal excitability are largely contributing to the reduction in SAI during movement. One behavioural reason for the increase in spinal excitability may be to reduce the inhibitory effects of short-latency somatosensory input on M1 because such inhibition may interfere with the ongoing movement. In support of this suggestion SAI is reduced in muscles involved in the movement but is increased in muscles not involved [23].

An important and novel finding was the reduction of SAI in the pre-movement component which occurs without changes in spinal excitability. Past research has indicated that SICI is reduced during the delay period between the ‘warning’ and ‘go’ cue in a choice reaction time task [26], [27] although SICI represents different inhibitory circuitry [16]–[20]. One mechanism for reduced SAI during the pre-movement component may relate to SEP gating as seen in monkeys during the delay period between a ‘warning’ and ‘go’ cue [28]. SEPs during the pre-movement component are reduced in M1 but unchanged at the level of the spinal cord or SI [28]. These data suggest that reduced SAI in the pre-movement component may be due to somatosensory input providing less inhibition on M1 corticospinal output, indicating a cortical origin of the reduced SAI in this movement component. The evidence from our study does not exclude the possibility that increases in spinal excitability may contribute to reduced SAI since F-waves may not represent the same pool of spinal motorneurons recruited by a TMS pulse [42]. However, our data suggest that reduced SAI in the pre-movement component is largely cortically mediated since F-waves remained unchanged. Specifically, it has been suggested that alterations in I3 wave can mediate large non-linear changes in MEP amplitude and we suggest that increases in this I wave created the observed differences in pre-movement SAI with minimal or no changes in spinal excitability [43].

There were similarities and differences in the degree of SAI evoked with cutaneous versus mixed nerve stimulation. The degree of SAI observed during rest was consistent with past studies using mixed [8], [10], [20], [22], [44], [45] and cutaneous [8], [23], [24] nerve stimulation. When comparing nerves, we observed an ∼35% increase in SAI for the mixed in relation to cutaneous nerve evoked SAI at rest. Increased SAI in the mixed versus cutaneous nerve has been observed in some instances [46] but not others [8]. We observed that SAI magnitude was greater in the pre-movement and tonic components for the mixed compared to cutaneous nerve, but the difference between nerves disappeared in the phasic component. This finding is different from a previous study demonstrating that SAI reduces MEPs by ∼50% for both the digital and median nerve stimulation during tonic contraction [8]. However, the latter difference may relate to the fact that the cutaneous nerve of both the 2^nd^ and 3^rd^ digit was stimulated [8]. The varying composition and volume of afferents recruited following stimulation of the median versus digital nerve may account for differences in SAI observed during rest and movement. Specifically, the larger volume of afferent input from the mixed nerve may have been driving the differences in SAI between the three movement components (rest, pre-movement, tonic). However, it does not account for the lack of difference between the nerves in the phasic component. One possibility is that nerve-specific differences in SAI depend on the relevancy of the afferent input to the ongoing movement though further research needs to explore this issue.

We tested whether the same results of movement-related modulation of SAI could occur when using a technically easier methodology of obtaining TMS intensities. Past research has used a standardized TMS output based on RMT for comparison of SAI during movement [13], [23], [24]. In the present study, we compared unconditioned MEPs evoked using a TMS intensity of 1.2 RMT and a TMS intensity normalized to produce ∼1 mV for each movement component. One disadvantage of using a standardized 1.2 RMT across all components of movement is that corticospinal excitability may be substantially different during movement (i.e., phasic, tonic) due to voluntary contraction [47]. We demonstrate that both approaches yield similar effects and suggest that a standardized TMS intensity is suitable for comparing SAI during movement to rest. However, when measuring subtle differences across movement components the two approaches yielded slightly different results. Specifically, there was more SAI in the tonic component for the ∼1 mV normalization. Additionally, MEP amplitudes during movement with a standardized TMS intensity based on RMT confounded the SAI results, as with this methodology greater reduction of SAI correlated with larger MEP amplitudes. We therefore suggest a TMS output based on a ∼1 mV normalization is a more suitable approach when comparing subtle differences across movement components compared to a normalization based on RMT as used elsewhere [13], [23], [24].

Somatosensory input is crucial for performing precise movements with the arm and hand. Inputs from the periphery can modulate corticospinal excitability depending on the time course of the input [20], [46], whether the inputs are natural [48] or electric [8], relevant to performing [49] and initiating a task [50]–[52] and following 40 minutes of repetitive ulnar nerve stimulation [53]. Our study is the first to compare SAI across different movement components and supports these finding such that short-latency somatosensory input from the periphery is modulated differently before and during movement and may be dependent on the composition or volume of afferent input carried by the stimulated nerve. This work may be applicable to certain movement disorders. SAI is altered in Parkinson's disease [54], in individuals with cerebellar symptoms [55], and after 1 Hz rTMS over SI in Writer's cramps [56]. SAI has also been shown to correlate with functional recovery from stroke such that a reduction in SAI is indicative of positive functional outcome [25]. It is evident that altered SAI is present in a number of movement disorders, but all of the aforementioned studies tested SAI at rest. Future studies in clinical populations may investigate the modulation of SAI during different components of movement to determine if ineffective SAI modulation is one factor contributing to motor symptoms.
